# Lacunar infarction aggravates the cognitive deficit in the elderly with white matter lesion

**DOI:** 10.1515/biol-2022-0027

**Published:** 2022-03-24

**Authors:** Wenjun Hu, Xing Guo, Yifeng Du

**Affiliations:** Department of General Practice, Central Hospital Affiliated to Shandong First Medical University, Jinan, Shandong Province 250013, China; Department of Neurosurgery, Qilu Hospital, Cheeloo College of Medicine, Shandong University, Jinan, Shandong Province, China; Department of Neurology, Shandong Provincial Hospital Affiliated to Shandong University, Jinan, Shandong, China

**Keywords:** white matter lesion, lacunar infarction, cognitive deficit, elderly

## Abstract

Cerebral white matter lesion (WML) and lacunar infarction (LI) were primary causes of cognitive deficit. Our study aimed to investigate the correlation between LI and cognitive deficit in the elderly with WML. A total of 118 participants (96 WML patients and 22 controls) were consecutively enrolled according to neuroimaging diagnosis of magnetic resonance imaging for this retrospective study. Neuroimaging evaluation and cognitive function assessment were analyzed. Compared with the controls, moderate and severe WML groups had significantly lower scores of Mini-mental State Examination (MMSE) and Montreal Cognitive Assessment (MOCA). Most cognitive domains of MOCA scores decreased, corresponding to the severity of WMLs. While there was no significant difference in score of MMSE between deep WML (DWML) and periventricular WML (PVL) groups, the scores of visuospatial/executive and naming function domains of MOCA appeared to be low in the DWML group. The scores of MMSE and MOCA were higher in only WMLs (WML−) group than WMLs combined with LIs (WML+) group, except for the naming cognitive domain. Moreover, LIs were independently correlated with the cognitive deficit in the elderly with WMLs. In the elderly with WMLs, the presence of LIs is associated with further aggravation of cognitive deficit.

## 1 Introduction

White matter lesions (WMLs) are common age-related changes among adults aged 60 years or older, and they can be observed on magnetic resonance imaging (MRI) [1]. It is well recognized that WML is an important risk factor for cognitive impairment [2,3]. Generally, WMLs are classified into periventricular WMLs (PVLs) adjacent to the ventricle and deep WMLs (DWMLs) separate from the ventricle according to MRI. Although WML emerges to be a predictor of cognitive deficit in the elderly, the independent association between the distribution and severity of WMLs and cognitive performance is inconsistent among studies.

Cerebral small vessel disease (CSVD) is the most frequent pathological neurological process that affects the brain’s small vessels and is a primary cause of cognitive deficit among the elderly [4,5]. WML and lacunar infarction (LI), which can be easily assessed by neuroimaging, are the main consequences of CSVD and often coincide. Previously, a well-characterized cohort study had showed that half of LI patients presented cognitive deficit [6]. However, it is still unclear whether the appearance of LI aggregates the impact of WMLs on cognitive performance among the elderly.

This study explained the correlation of WMLs at different severity and location with a cognitive deficit in the elderly. Furthermore, we explored the relationship between WMLs combined with LIs and cognitive deficit.

## 2 Materials and methods

### 2.1 Study population

One hundred and eighteen participants (age range between 60 and 85 years, 75.4 ± 5.54 years) were recruited in Shandong Provincial Hospital, Shandong University, China. Among the participants, 96 patients with WMLs, diagnosed by neuroimaging evaluation, were included from the Department of Neurology. Besides, 22 participants who passed medical health examinations, including neuroimaging evaluation, to exclude the existence of WMLs and LIs were considered controls. The controls were matched with WML patients in clinical characteristics.


**Inclusion criteria:** (i) All participants included in this study met the diagnostic criteria according to the neuroimaging evaluation. (ii) The participants’ age ranged from 60 to 85 years old. (iii) There was no medication history affecting cognition. (iv) All participants were right-handed.
**Exclusion criteria:** (i) Participants with large area cerebral infarction, acute cerebral hemorrhage, previous cerebral hemorrhage history, or traumatic brain injury. (ii) Participants with serious heart, liver, or kidney organ dysfunction. (iii) Participants with mental illness, neurocognitive impairment caused by depression or Binswanger disease. (iv) Participants with drug or alcohol abuse.
**Informed consent:** Informed consent has been obtained from all individuals included in this study.
**Ethical approval:** The research related to human use has been complied with all the relevant national regulations, institutional policies and in accordance with the tenets of the Helsinki Declaration, and has been approved by the ethics committee of Shandong Provincial Hospital.

### 2.2 Neuroimaging evaluation

In this study, all participants were scanned using a SIGNA HD 3.0-Tesla superconducting MRI system of GE Company. We collected the cranial MRI images consisting of T1-weighted image (T1WI), T2-weighted image (T2WI), fluid-attenuated inversion recovery image (FLAIR). The acquired images were visually assessed by two experienced radiologists without knowledge of the clinical data.

Neuroimaging evaluations of WMLs and LIs were performed according to the conventional structural MRI techniques. WML was defined as isointensity or hypointensity on T1WI and hyperintensity on T2WI, FLAIR in periventricular or subcortical deep white matter [7]. LI was defined as hypointensity on T1WI, clear boundary hyperintensity on T2WI with a diameter of 2–15 mm, and surrounded by a hyperintense rim on FLAIR [8]. According to the neuroimaging evaluations, the WML patients were divided into control group (*n* = 22), only WML (WML−) group (*n* = 70), and WML combined with LI (WML+) group (*n* = 26), respectively.

WML− were divided into periventricular WMLs (PVLs, *n* = 39) and deep WMLs (DWMLs, *n* = 31). The severity of PVLs and DWMLs was classified separately using Fazekas’s rating scale into 1–3 grades, referring to mild, moderate, and severe [9]. PVLs were graded as follows: grade 1, caps or pencil-thin lining lesions; grade 2, smooth halo lesions; grade 3, irregular lesion extending into the deep white matter. DWMLs were graded as follows: grade 1, punctate foci; grade 2, beginning confluence of punctate or patchy foci; and grade 3, large confluent areas. In terms of WML− groups, patients were divided into mild WML group (*n* = 30), moderate WML group (*n* = 20), and severe WML group (*n* = 20).

### 2.3 Neuropsychological tests

All participants had completed an extensive neuropsychological test battery. The detection indicators included the clinical data (age, gender, education years, blood pressure, blood glucose, blood lipid, heart disease, smoking, drinking), neuropsychiatric inventory [Montreal Cognitive Assessment (MOCA), and Mini-mental State Examination (MMSE)]. All participants were instructed to complete the total score of MMSE [10]. MOCA was conducted to evaluate the cognitive function in eight domains, including visuospatial/executive function, memory, naming, language, attention/calculation delayed recall, abstract thinking, and orientation. Affected by the education years, one point should be added to the total score for subjects who were educated less than 12 years.

### 2.4 Statistical analyses

Data analyses were conducted with SPSS 22.0 (SPSS, IL, USA). The continuous variable of normal distribution was presented as mean ± standard deviation, and data were analyzed using one-way analysis of variance (ANOVA) for comparison between multiple groups, Student’s 2-tailed *t*-test for that between two groups. The classified variable was expressed as frequency or proportion, and χ 2 test was applied to compare groups. Eta coefficient was performed to analyze the correlation between scores of MMSE and MOCA and the severity of WMLs. In comparison to the WML− group with WML+ group, analyses of MMSE and MOCA were adjusted for WMLs severity, age, and education by multiple linear regression. All tests were 2-tailed, and *P-*value <0.05 was considered as a statistically significant difference.

## 3 Results

### 3.1 Baseline clinical characteristics of the study samples

In this study, there were 96 WML patients with or without LI and 22 matched controls. Ninety-six patients were divided into two groups of WML− group (*n* = 70) and WML+ group (*n* = 26). According to the severity of WMLs, WML− group included three subgroups as follows: mild WML group (*n* = 30), moderate group (*n* = 20), and severe group (*n* = 20). The baselines of demographic and clinical characteristics had no significant statistical difference among matched control group, mild WML group, moderate WML group, severe group, and WML+ group (Table 1). We further compared the baselines of clinical characteristics between WML− group and WML+ group, and WML+ group exhibited more severity of WML (Table 2).

**Table 1:** Clinical characteristic of 118 subjects

Variables	Controls	WML−			WML+	*F*/*χ* ^2^	*P* value
		Mild	Moderate	Severe			
*N*	22	30	20	20	26		
Sex (male/female)	12/10	17/13	13/7	12/8	15/11	0.557	0.968
Age (years)	74.55 ± 6.4	74.57 ± 5.4	75.45 ± 5.3	77.05 ± 4.6	76.00 ± 5.7	0.812	0.520
Education (y/n) (≥12 years)	12/10	14/16	9/11	10/10	9/17	2.145	0.709
Hypertension (y/n)	4/18	10/20	5/15	6/14	4/22	3.231	0.520
Hyperlipidemia (y/n)	3/19	4/26	4/16	7/13	10/16	7.705	0.103
Heart disease (y/n)	1/21	2/28	3/17	3/17	5/21	3.627	0.459
Type 2 diabetes (y/n)	3/19	4/26	8/12	6/14	10/16	6.724	0.075
Current smoker (y/n)	6/16	9/21	7/13	11/9	11/15	4.633	0.327
Current drinker (y/n)	2/20	7/23	8/12	4/16	7/19	5.833	0.212

WMLs− = only white matter lesions, WML+ = white matter lesions combined with lacunar infarctions.

**Table 2:** Clinical characteristics of only WMLs and WML–LIs subjects

Variables	WMLs		*t*/*χ* ^2^	*P* value
	WML−	WML+		
*N*	70	26		
Sex (male/female)	42/28	15/11	0.042	0.838
Age (years)	75.45 ± 5.5	76.00 ± 5.7	−0.366	0.908
Education (y/n) (≥12 years)	33/37	9/17	1.209	0.272
Hypertension (y/n)	21/49	4/22	2.103	0.147
Hyperlipidemia (y/n)	15/55	10/16	2.856	0.091
Heart disease (y/n)	8/62	5/21	0.986	0.321
Type 2 diabetes (y/n)	18/52	10/16	1.491	0.222
Current smoker (y/n)	27/43	11/15	0.111	0.739
Current drinker (y/n)	19/51	7/19	0.001	0.983
WMLs severity			6.341	0.042
Mild (*n*, %)	30(42.8)	5(19.2)		
Moderate (*n*, %)	20(28.6)	7(26.9)		
Severe (*n*, %)	20(28.6)	14(53.9)		

WMLs = white matter lesions, WMLs− = only white matter lesions, WML+ = white matter lesions combined with lacunar infarctions.

### 3.2 Correlation between severity of WMLs and cognitive function in the elderly

**Figure 1:**
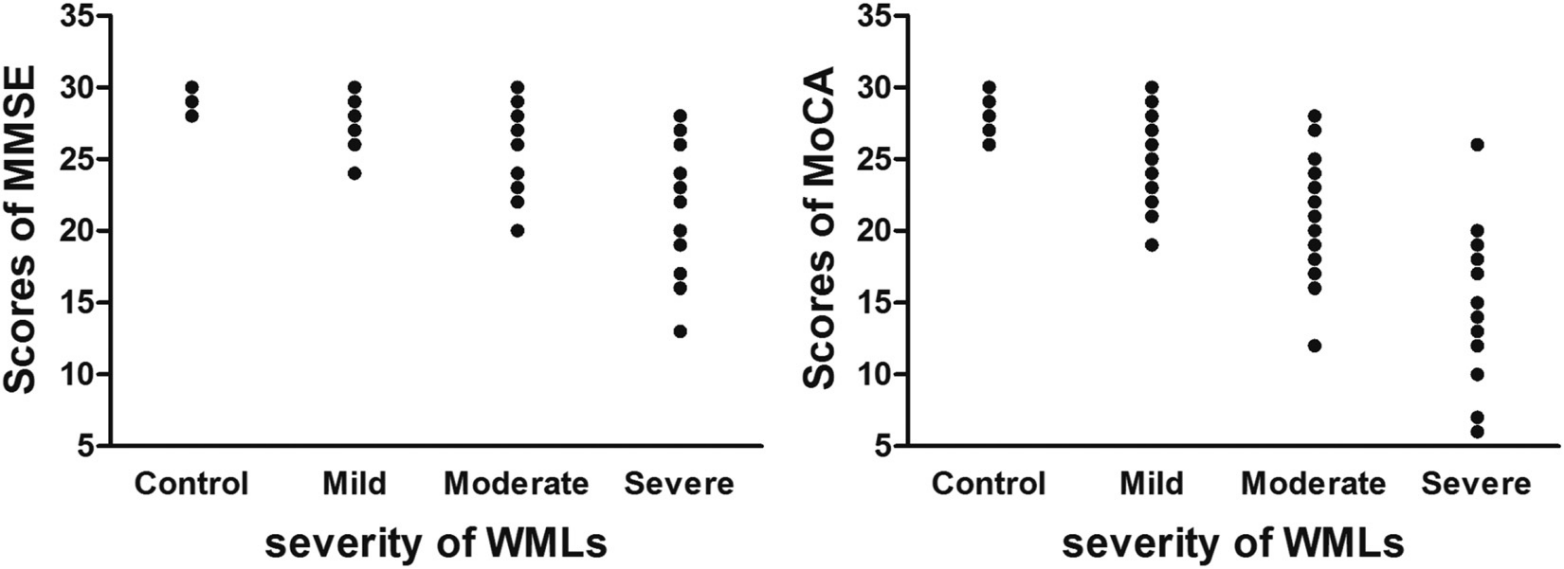
Scatter plot of relationship between the severity of WMLs and cognitive function assessed by MMSE and MOCA.

By comparing with control group and mild WML group, moderate WML group and severe WML group had significantly lower scores in total scores of MMSE and MOCA. Only the visuospatial/executive cognitive domain score had a significant difference between the control group and the mild WML group. The severe WML group had lower scores than the moderate WML group in visuospatial/executive function scores, attention/calculation, language, abstract thinking, and memory cognitive domains (Table 3). More importantly, a scatter plot showed that total scores of MMSE and MOCA negatively correlated with the severity of WMLs (Figure 1 MOCA Eta^2^ = 0.638, *P* < 0.001; MMSE Eta^2^ = 0.596, *P* < 0.001), indicating that cognitive function impaired corresponding to increase in severity of WMLs.

**Table 3:** Association between severity of WMLs and cognitive function

	Controls	WML−			*F*	§*P* value
		Mild	Moderate	Severe		
*N*	22	30	20	20		
MMSE	29.27 ± 0.70	27.90 ± 1.75	26.10 ± 2.73*^,†^	21.15 ± 3.99*^,†,‡^	43.29	<0.001
MOCA	27.59 ± 1.09	25.97 ± 2.55	20.85 ± 3.92*^,†^	15.45 ± 5.65*^,†,‡^	51.62	<0.001
Visuospatial/executive function	4.45 ± 0.59	4.01 ± 0.85*	2.65 ± 1.31*^,†^	1.45 ± 1.19*^,†,‡^	37.04	<0.001
Naming	2.91 ± 0.29	2.97 ± 0.18	2.45 ± 0.61	2.20 ± 0.89*^,†^	10.92	<0.001
Language	2.73 ± 0.46	2.67 ± 0.55	2.40 ± 0.77*	1.25 ± 0.85*^,†,‡^	23.35	<0.001
Abstract thinking	1.50 ± 0.51	1.43 ± 0.68	1.25 ± 0.64	0.70 ± 0.80*^,†,‡^	6.37	0.001
Delayed recall	4.55 ± 0.51	4.30 ± 0.70	3.55 ± 1.15*^,†^	2.20 ± 1.24*^,†,‡^	28.51	<0.001
Orientation	5.86 ± 0.35	5.47 ± 0.78	4.70 ± 1.08*^,†^	4.30 ± 1.17*^,†^	14.01	<0.001
Attention/calculation	5.68 ± 0.65	5.37 ± 1.13	4.10 ± 1.07*^,†^	3.25 ± 1.86*^,†,‡^	18.44	<0.001

WMLs− = only white matter lesions, MMSE = mini-mental state examination, MoCA = montreal cognitive assessment.

*Compared with normal control, *P* < 0.05.

^†^Compared with moderate WML, *P* < 0.05.

^‡^Compared with severe WML, *P* < 0.05.

§*P* value of analysis of variance by one-way ANOVA.

### 3.3 Correlation between location of WMLs and cognitive function in the elderly

Compared with the control group, both PVL and WML groups had significantly lower total scores of MMSE and MOCA, as well as the scores of visuospatial/executive function, language, delayed recall, orientation, and attention/calculation cognitive domains. However, there were no differences in total MMES and MOCA scores between the PVL and DWML groups. Among cognitive domains, the scores of visuospatial/executive function and naming were lower in the DWML group than that in the PVL group, indicating that DWMLs have a more significant effect on visuospatial/executive and naming impairment than with PVLs (Table 4).

**Table 4:** Association between location of WMLs and cognitive function

	Controls	WML−		*F*	^‡^ *P* value
		PVLs	DWMLs		
*N*	22	39	31		
MMSE	29.27 ± 0.70	25.5 ± 4.16*	25.3 ± 3.90*	9.86	<0.001
MOCA	27.59 ± 1.09	22.2 ± 5.92*	20.5 ± 5.90*	12.44	<0.001
Visuospatial/executive function	4.45 ± 0.60	3.18 ± 1.32*	2.29 ± 1.49*^,†^	19.22	<0.001
Naming	2.91 ± 0.29	2.74 ± 0.54	2.42 ± 0.86*^,†^	4.93	<0.001
Language	2.73 ± 0.46	2.31 ± 0.83*	1.90 ± 0.98*	6.61	0.002
Abstract thinking	1.50 ± 0.51	1.26 ± 0.75	1.06 ± 0.77	2.43	0.094
Delayed recall	4.55 ± 0.51	3.56 ± 1.35*	3.65 ± 1.31*	7.17	0.001
Orientation	5.86 ± 0.35	5.03 ± 1.06*	4.77 ± 1.15*	8.47	<0.001
Attention/calculation	5.68 ± 0.65	4.36 ± 1.77*	4.45 ± 1.43*	6.50	0.002

WMLs− = only white matter lesions, PVLs = peri-ventricular white matter lesions, DWMLs = deep white matter lesions, MMSE = mini-mental state examination, MoCA = montreal cognitive assessment.

*Compared with normal control, *P* < 0.05.

^†^Compared with WML–PVL, *P* < 0.05.

^‡^ *P* value of analysis of variance by one-way ANOVA.

### 3.4 Comparison of cognitive function between WML group and WML+ group

The WML+ group had notably lower total scores of MMSE and MOCA and scores of all cognitive domains than the control group, while the WML− group had a lower total score of MOCA and scores of visuospatial/executive, delayed recall, orientation, and attention/calculation than the control group. The total scores of MMSE and MOCA and most scores of the cognitive domains were higher in WML− group than WML+ group, except for naming the cognitive domain (Table 5). To clarify whether LIs in the elderly with WMLs were an independent factor of cognitive function, we performed multiple linear regression. Although WML− severity had statistical difference between WML− and WML+ groups, age and education were involved in multiple linear regression due to close correlation with cognitive function [11]. On the multivariate linear regression analysis, using variables of age, education, and WMLs severity, we confirmed that LIs were independently correlated with the cognitive function in the elderly with WMLs (Table 6).

**Table 5:** Comparison of cognitive function between WML group and WML–LI group

	Controls	WML−	WML+	*F*	^‡^ *P* value
*N*	22	70	26		
MMSE	29.27 ± 0.70	25.4 ± 3.98	20.6 ± 3.64*^,†^	46.04	<0.001
MOCA	27.59 ± 1.09	21.5 ± 5.93*	14.9 ± 5.15*^,†^	35.62	<0.001
Visuospatial/executive function	4.45 ± 0.60	2.79 ± 1.45*	1.77 ± 1.28*^,†^	25.88	<0.001
Naming	2.91 ± 0.29	2.60 ± 0.67	2.42 ± 0.76*	3.52	0.033
Language	2.73 ± 0.46	2.13 ± 0.92	1.58 ± 0.99*^,†^	10.49	<0.001
Abstract thinking	1.50 ± 0.51	1.17 ± 0.76	0.46 ± 0.58*^,†^	15.37	<0.001
Delayed recall	4.55 ± 0.51	3.49 ± 1.33*	2.08 ± 1.16*^,†^	26.75	<0.001
Orientation	5.86 ± 0.35	4.91 ± 1.10*	4.19 ± 1.36*^,†^	14.51	<0.001
Attention/calculation	5.68 ± 0.65	4.40 ± 1.62*	2.35 ± 1.67*^,†^	31.12	<0.001

WMLs− = only white matter lesions, WML+ = white matter lesions combined with lacunar infarctions. MMSE = mini-mental state examination, MoCA = montreal cognitive assessment.

*Compared with normal controls, *P* < 0.05.

^†^Compared with only WMLs, *P* < 0.05.

^‡^ *P* value of analysis of variance by one-way ANOVA.

**Table 6:** Multiple linear regression analysis for the relationship between cognitive function and potential confounding variables

Variables	MMSE		MOCA	
	*t*	*P* value	*t*	*P* value
WMLs with or without LIs	−5.818	<0.001	−4.229	<0.001
WML severity	−6.827	<0.001	−7.135	<0.001
Age	1.546	0.126	1.235	0.220
Education	0.328	0.744	1.134	0.260

WMLs = white matter lesions, Lis = lacunar infarctions, MMSE = mini-mental state examination, MoCA = montreal cognitive assessment.

Data are *t* (*P* value) of the correlation.

MMSE *r* ^2^ = 0.522; MOCA *r* ^2^ = 0.474.

## 4 Discussion

Cognitive deficit is the predominant reason for the burden of aging society around the globe [12]. Therefore, an early diagnosis and intervention by physicians are critical in the elderly with cognitive deficit [13]. In this study of the elderly, we verified a close association of different severity and location of WMLs with the cognitive deficit, including various cognitive domains. Moreover, we confirmed that LIs were an independent factor influencing cognitive impairment in the elderly with WMLs.

The two scales of MOCA and MMSE were used to comprehensively evaluate cognitive function. We found that the average total scores of MOCA of subjects were significantly lower than those of MMSE. This result verified that the MOCA scale is more sensitive to the evaluation of cognitive deficit than the MMSE scale, which was the same as the former study [14]. In general, we found that the severity of WMLs was negatively correlated with cognitive function, including visuospatial/executive function, delayed recall, orientation, and attention/calculation. Only visuospatial/executive function was declined in mild WML groups, indicating that early dysfunction occurred in visuospatial/executive function. In contrast, severe WMLs caused a mild impairment in cognitive performance in the elderly, consistent with previous research [15]. Our results demonstrated that cognitive deficit happened with the progress of WML severity.

Scholars believed that the severity of cognitive impairment in WML patients was related to the location of WML. The purpose of this study was also to explore the different effects of the location of WMLs on the cognitive deficit. We found that there was no difference in total scores of MMSE and MOCA between PVL and DWML. Meanwhile, the DWMLs had more significant effects on cognitive domains of visuospatial/executive and naming than PVLs. Previous studies showed that WMLs disrupted both short and long connections which deliver various information of cortical areas to regulate special or multiple domains of cognition [16,17]. DWMLs mainly influenced the function of the spatial brain region by damaging short connections. More importantly, visuospatial/executive function and naming were respectively related to multiple motor cortexes and temporal cortexes, so these two cognitive domains were more sensitive to DWMLs.

WMLs and LIs located in the subcortical structures were mainly forms of age-related CSVD [18,19]. A recent study demonstrated that LIs were associated with cognitive deficits caused by affecting the white matter tract integrity [20]. A study involving 639 subjects showed that WMLs and LIs were independent influencing factors of cognitive impairment [21]. Likewise, Benisty et al. reported that the LIs in different parts of the subcortical white matter had an important impact on cognitive impairment, independent of WMLs [22]. Although plenty of studies verified that LIs affected cognitive function, the clinical value of WMLs combined with LIs on cognitive performance in the elderly was still not clear. Our study found that compared with WML− group, WML+ group had significantly low scores in all cognitive domains of MOCA, except for naming the cognitive domain. In addition, after being adjusted by variables of WML severity, age and education, LIs were independently associated with cognitive dysfunction in the elderly with WMLs. Thus, our results indicated that WMLs combined with LIs could deteriorate cognitive impairment in the elderly.

This study highlighted a detailed description of the influence of WMLs with or without LIs on the cognitive deficit in the elderly, which provided a theoretical basis for prediction and early medical intervention of cognitive impairment. However, there are several limitations to our observational study. The sample size in this study is limited. Moreover, as the association between location and number of LIs and cognitive deficit were not analyzed, the impact of WMLs and LIs on cognitive function remains to be further studied.
